# Partial compliance with symptom-based testing pathways reveals asymptomatic carriage of SARS-CoV-2 in Ireland

**DOI:** 10.1007/s11845-020-02375-4

**Published:** 2020-09-28

**Authors:** Grace Yixin Chan, Lillian Rajan, Cillian De Gascun, Niamh O’Flaherty

**Affiliations:** 1 UCD National Virus Reference Laboratory, University College Dublin, Belfield, Dublin 4, Ireland; 2 Irish Blood Transfusion Service, National Blood Centre, James’s Street, Dublin 8, Ireland

## Introduction

On 29 February 2020, approximately 3 months after the discovery of a novel human coronavirus in China, Ireland reported its first case of COVID-19 caused by severe acute respiratory syndrome coronavirus 2 (SARS-CoV-2). Ten days later, a pandemic was declared by the World Health Organisation (WHO). Since that time, over 9.6 million cases have been reported worldwide. As of 27 June 2020, 25,414 COVID-19 cases and 1730 deaths have been notified in Ireland [1].

In response to this crisis, the National Virus Reference laboratory (NVRL) introduced a laboratory-developed real-time RT-PCR assay targeting the E gene and RdRp gene per Corman et al*.* [2]. Subsequently, a commercial assay RealStar® SARS-CoV-2 RT-PCR Kit 1.0 (Altona Diagnostics, Hamburg, Germany), designed for the detection of the B-βCoV (target E gene) and SARS-CoV-2 (target S gene) RNA was introduced.

In the initial stages of the outbreak, patients with suspected SARS-CoV-2 infection were transported by the National Ambulance Service (NAS) to hospitals with appropriate isolation facilities where respiratory samples were obtained and the patient awaited test results onsite. As the epidemiology of the outbreak changed from mostly European travel–related cases to local community transmission, the NAS commenced sampling of stable symptomatic individuals for SARS-CoV-2 in their home. Community sampling now takes place in one of 46 largescale ‘sampling hubs’ around the country as directed by public health, primary care and infection specialists according to national guidance [3]. This novel process has greatly facilitated the detection, isolation and contact tracing of SARS-CoV-2 infection in Ireland.

The focus of this report is to review the clinical presentation of early COVID-19 cases tested by NAS in the community and reported by the NVRL, during the 2nd week of March 2020, with particular reference to national guidelines.

## Methods

The first 100 laboratory-confirmed COVID-19 cases in Ireland whose respiratory samples were obtained in the community and tested in the NVRL were retrospectively reviewed. To estimate the agreement with national guidance, we used Versions 2 and 3 of the *‘Telephone assessment and testing pathway for patients who phone general practice and healthcare settings other than receiving hospitals’* approved by the COVID-19 Expert Advisory Group of the National Public Health Emergency Team and issued by the Health Protection Surveillance Centre (HPSC). Version 8 was also included for comparison as this was the testing pathway active at the time of writing [4] (Table 1). Using the NVRL Laboratory Information System which includes a scanned copy of the test request form, the clinical, travel, relevant COVID-19 contact history and the cycle threshold (Ct) values of the real-time RT-PCR results were recorded on a database using Microsoft Excel. These data were irrevocably anonymised before analysis.

**Table 1:** COVID-19 assessment and testing pathways in the community: clinical and epidemiological criteria

Version 2: 1. Acute mild respiratory infection (including one of: fever, shortness of breath or cough) OR fever of unknown cause with no other symptoms. AND 2. In the 14 days before onset of illness: been in an area with presumed community transmission of COVID-19, OR contact with a case of COVID-19, OR worked in or attended a healthcare facility abroad where patients with COVID-19 being treated.	
Version 3: (clinical criteria only) Recent onset of fever or chills AND/OR symptoms of respiratory tract infection, which includes cough.	
Version 8: 1. Acute respiratory infection (sudden onset of at least one of: cough, fever, shortness of breath) AND no other aetiology that fully explains the clinical presentation. OR 2. Acute respiratory illness AND having been in close contact with a confirmed or probable COVID-19 case in the last 14 days prior to onset of symptoms. A probable case is one who meets criterion 1 but has not been tested. Adapted from HPSC [4].	

Descriptive statistics were used. Categorical variables were expressed in numbers (percentage); continuous variables were expressed using median with range or interquartile range (IQR) and compared with Mann-Whitney *U* test. Statistical analysis was performed using SPSS (IBM SPSS, Armonk, New York, USA). *P* value of < 0.05 was regarded as statistically significant.

## Results

### Clinical characteristics

**Fig. 1:**
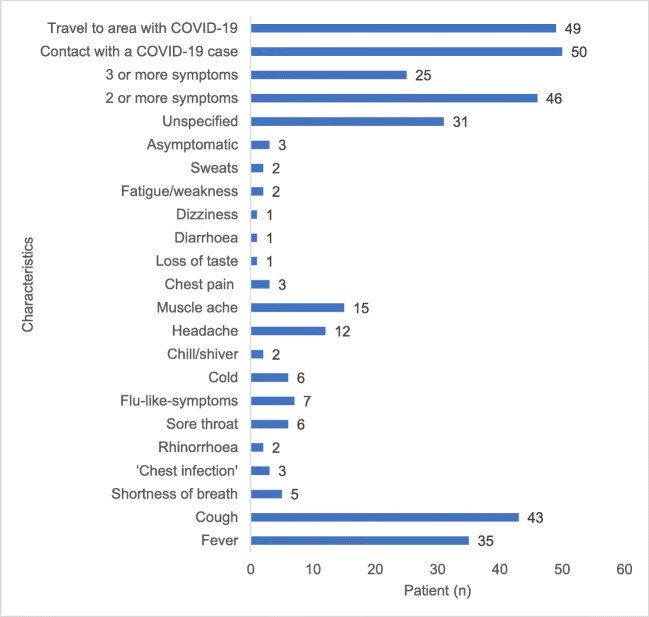
Characteristics of laboratory-confirmed community COVID-19 cases, Ireland, March 2020 (*n* = 100)

Sixty percent (60/100) of the laboratory-confirmed COVID-19 cases were reported in the east of the country, particularly in Dublin, where 44% of Ireland’s urban population reside [5]. The median age was 40.5 (IQR 27–56). The most common clinical features were cough, fever, muscle ache, and headache (Fig. 1). Night sweats, loss of taste in the absence of respiratory features (*n* = 2) and GI symptoms (*n* = 1) were uncommon characteristics. The date of onset of symptoms was documented in 71/100 (71%) of cases. The median duration of symptoms prior to testing was 4 days (range 1–13 days). Overall, 66/100 (66%) of the COVID-19 cases we identified in the community were reported to be symptomatic. Their clinical presentations were broadly aligned with the national testing pathways. In the symptomatic group, the clinical features prompting testing indicated compliance with testing criteria in 77% (51/66) of cases at the beginning of the outbreak and reaching 95% (63/66) with the version of the testing pathway in use at the time of writing (Table 2).

**Table 2:** Clinical characteristics of symptomatic COVID-19 cases and the proportion of cases fulfilling the clinical criteria for testing in the community, Ireland, March 2020 (*n* = 66)

Clinical characteristics of symptomatic patients	*n* (%)	Compliance rate with HPSC guidelines at the time of:		
		Testing		Review
		Ver 2^a,b^	Ver 3^c^	Ver 8^d^
Fever, cough or shortness of breath:	58 (87.9)	51	58	58
1 out of 3 symptoms	35			
2 out of 3 symptoms	21			
3 out of 3 symptoms	2			
Respiratory symptoms other than fever, cough or shortness of breath	5 (7.6)		5	5
Other non-respiratory symptoms only:	3 (4.5)			
Headache	1			
Night sweats	1			
Loss of taste	1			
Total, *n* (%)	66 (100)	51 (77)	63 (95)	63 (95)

*HPSC*, Health Protection Surveillance Centre; *Ver*, version

^a^In the last 14 days prior to onset of symptoms

^b^Acute mild respiratory infection (cough, fever or shortness of breath) AND in the 14 days before onset been in an area with presumed COVID-19 or contact with a case

^c^Recent onset of fever or chills AND/OR symptoms of respiratory tract infection, which includes cough

^d^Acute respiratory infection (cough, fever or shortness of breath) and no other explanation OR acute respiratory illness and close contact with a confirmed/probable case

### SARS-CoV-2 PCR characteristics

**Fig. 2:**
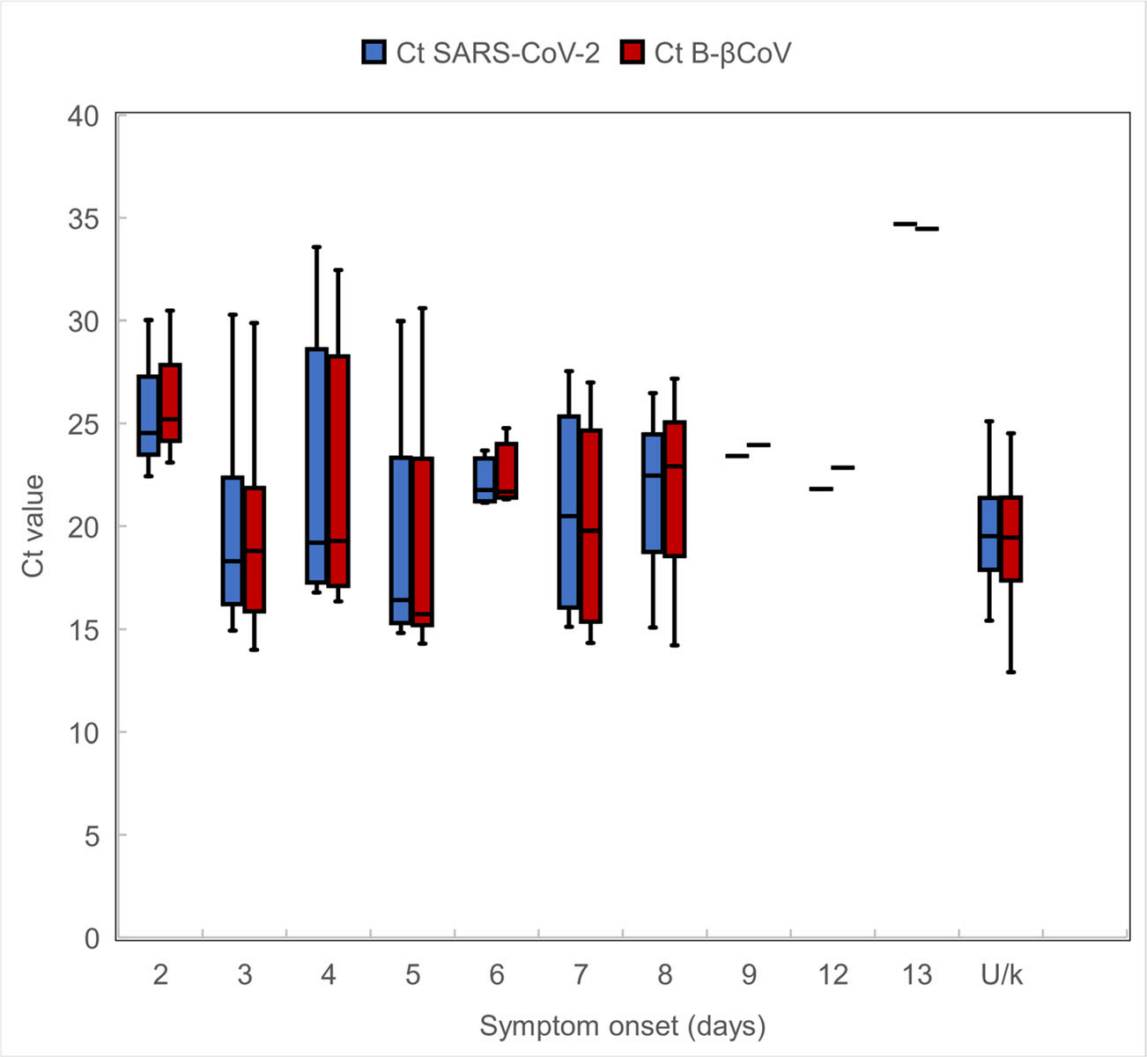
The Ct values for B-βCoV and SARS-CoV-2 specific RNAs in relation to symptom onset, Ireland, March 2020 (*n* = 100). Ct, cycle threshold; B-βCoV, B-betacoronavirus; U/k, unknown. Significant difference was not observed in the Ct values for B-βCoV (*P* = 0.498) or SARS-CoV-2 specific RNA (*P* = 0.565) for cases with symptom onset ≤ 7 days and those with > 7 days onset as determined by Mann-Whitney *U* test

Upper respiratory samples were tested using the commercial RealStar® SARS-CoV-2 RT-PCR Kit 1.0 (Altona Diagnostics, Hamburg, Germany) with specific probes for lineage B-betacoronavirus (B-βCoV) and SARS-CoV-2 specific RNAs. The median Ct value for the B-βCoV specific RNA (target E gene) was 20 (range 12.9–34.5). The median Ct value of SARS-CoV-2 specific RNA (target S gene) was 19.9 (range 12.6–34.7). There was no statistical difference in the Ct values for B-βCoV (*P* = 0.498) or SARS-CoV-2 specific RNA (*P* = 0.565) for cases with symptom onset within 7 days of being tested and those with symptoms for more than 7 days before a sample was obtained (Fig. 2).

## Discussion

The clinical features of cough, fever, myalgia, headache as well as a constellation of other respiratory and non-respiratory features reflect the description of COVID-19 reported in the literature [6, 7]. Although 46/100 (46%) of patients had more than one symptom, only 2/100 (2%) had all 3 classic features of COVID-19, i.e. cough, fever and shortness of breath. This is not unexpected in a group who did not attend emergency services. In one third of cases (31/100), symptoms were not documented; however, 6 out of 31 (19%) of these cases did report a relevant travel and/or contact history. Further iterations of the guidance and expansion of case definitions (e.g. that did not require clinical AND epidemiological features) led to increased compliance with testing pathways. Interestingly, 3/100 (3%) individuals who reported feeling well at the time of testing were close contacts of known COVID-19 cases. These cases indicated that asymptomatic infections were present in the Irish community in the initial stages of the SARS-CoV-2 outbreak in Ireland.

RealStar® SARS-CoV-2 RT-PCR Kit has been proven to be a reliable assay [8] and was one of the most widely used in Ireland for the detection of SARS-CoV-2 RNA at the time of writing. We have found the performance of the assay to be satisfactory. The Ct values obtained for either B-βCoV or SARS-CoV-2 specific RNA of the RealStar® SARS-CoV-2 assay were less than 35, within the acceptable range according to the manufacturer’s instructions. Although one might have expected an association between viral load (inversely related to Ct value) in those tested soon after clinical onset versus those at a later stage in the illness, a trend was not observed in this regard (*P* > 0.05) (Fig. 2). A larger sample and a longer follow-up period might have identified such a trend as many reports indicate a high viral load at the early stage of illness [9–12]. Other factors such as missing data, sample quality and type, storage conditions and a lack of follow-up samples may have influenced this aspect of the review. Additionally, in comparing those with the classic features of cough, fever or shortness of breath and those without these features, the RT-PCR Ct values did not show a significant difference (*P* = 0.3) (data not shown).

We found a small number of individuals who presented without any respiratory features including one case with loss of taste. Sudden onset of loss of sense of smell, loss or distortion of sense of taste are now included in the clinical criteria of the case definition for COVID-19. Testing pathways are needed to allocate resources, to aid in the streamlining of testing, and ultimately to guide the identification of cases but the criteria are not all encompassing. Continuous review and revision of symptom-based case definitions is required as new clinical and epidemiological information becomes available to decision-makers tasked with the containment of SARS-CoV-2.

## Conclusion

Cough, fever, contact with a known COVID-19 case and travel to an affected region were the most common features reported by patients with laboratory-confirmed SARS-CoV-2 infection tested in the community. Headache, night sweats, loss of taste without respiratory features (*n* = 3) and GI symptoms (*n* = 1) were uncommon characteristics in this group. Although compliance with testing criteria was not 100%, Irish clinicians are afforded latitude in relation to identifying patients for SARS-CoV-2 testing. Indeed, we noted 3 patients who did not have discernible clinical symptoms of COVID-19, but who did have a positive test result. These cases were an early indicator of asymptomatic infection in the community which is one of the major challenges for containment of the virus [13, 14]. It was interesting to note that in the cohort presented here, viral loads (inferred from Ct values) were not significantly associated with the timing of the test (in relation to illness onset) or indeed the presence of classic respiratory symptoms (if any).
